# A rapid review of digital approaches for the participatory development of health-related interventions

**DOI:** 10.3389/fpubh.2024.1461422

**Published:** 2024-11-29

**Authors:** Friederike Doerwald, Imke Stalling, Carina Recke, Heide Busse, Rehana Shrestha, Stefan Rach, Karin Bammann

**Affiliations:** ^1^Institute for Public Health and Nursing Research (IPP), Working Group Epidemiology of Demographic Change, University of Bremen, Bremen, Germany; ^2^Leibniz ScienceCampus Digital Public Health Bremen, Bremen, Germany; ^3^Department of Prevention and Evaluation, Leibniz Institute for Prevention Research and Epidemiology - BIPS, Bremen, Germany; ^4^Department of Social Epidemiology, Institute for Public Health and Nursing Research (IPP), University of Bremen, Bremen, Germany; ^5^Department of Epidemiological Methods and Etiological Research, Leibniz Institute for Prevention Research and Epidemiology - BIPS, Bremen, Germany

**Keywords:** digital methods, action research, co-research, community participation, public health, health promotion

## Abstract

**Objectives:**

Using participatory approaches to design health interventions is promising, and the ongoing digitalization has enabled the development of diverse digital formats for this purpose. These digital formats bring forth distinct advantages and challenges that should be carefully considered. This rapid review aims to present an overview of digital formats employed in participatory health intervention development and their reported benefits and barriers.

**Design:**

A qualitative rapid review was conducted, following recommendations by the Cochrane Rapid Reviews Methods Group. The literature search was carried out in October 2022 and encompassed the PubMed, Embase, PsycINFO, and Cochrane CENTRAL databases. Studies were included if they were published in 2010 or later and reported the development of a health-related intervention employing digital formats in the participatory process.

**Results:**

A total of 22 studies were included. We identified three types of digital formats used for participatory health intervention development: web-based participatory formats (*n* = 14), digital participatory visual formats (*n* = 5), and digital participatory mapping (*n* = 3). The reported benefits of applying digital formats included enhanced participant anonymity, increased time and cost efficiency, and more flexibility regarding scheduling and extent of participation. Among the reported barriers were sufficient internet connectivity, required technical skills, and online fatigue.

**Conclusion:**

The review shows a variety of digital formats employed to develop participatory health interventions. Yet, these methods are primarily digital adaptations of pre-existing analog formats. Innovative digital approaches involving, for example, virtual reality devices remain largely unused. The review also revealed a need for establishing shared terminology and reporting standards to facilitate communication, comparison, and synthesis of findings in this evolving area of research.

## 1 Introduction

Participatory approaches for intervention development, that is, the active involvement of stakeholders of different levels, including the target group, in the intervention development process and the transfer of decision-making power to these stakeholders have been increasingly recognized as a promising way to increase the effectiveness of health interventions (1) and to address health disparities (2). Such approaches have, for instance, been used to develop interventions to promote physical activity in older adults (3), to improve mental health in adolescents (4), and to prevent HIV infections in adolescents (5). Digitalization has opened new doors for participatory approaches in public health research. Digital participatory approaches for intervention development employ digital technology, such as the internet, mobile applications, or virtual technology, for the participatory process. A scoping review by Schroeer et al. (6) on digital participatory approaches utilized in community-based participatory research, which included eleven studies, observed that a wide variety of digital formats, such as online focus groups, online forums, or online concept mapping, have been used. It is important to note that the aforementioned review by Schroeer et al. (6) included studies published until November 2020. The COVID-19 pandemic and its prevention measures (e.g., physical distancing) forced many researchers to switch their participatory research to an online environment, and, as a result, research in this field has quickly accumulated (7–9).

Digital participatory approaches to studying public health issues are, however, not only viable in pandemic times but generally have several putative benefits over analog participatory approaches. Digital participatory approaches, for instance, have the potential to reach groups that are unable or hesitant to participate in offline participatory research projects (10). Digital formats such as video conferencing can also be cost-effective, as they do not involve expenses for travel or hiring venues (11). Despite its benefits, digital participatory methods in public health research are often accompanied by structural barriers, such as access to the internet and high levels of computer literacy (12, 13). This highlights the need to carefully consider advantages and barriers when employing digital participatory approaches.

This rapid review aims to summarize the current state of the fast-evolving literature on health-related intervention development utilizing digital participatory approaches. We provide an overview of the digital formats used to participatively develop health-related interventions and their reported benefits as well as barriers to inform and guide researchers and practitioners. In contrast to the existing review by Schroeer et al. with its broader approach (6), the present rapid review focuses on digital participatory approaches that were used for the *development* of health interventions. More specifically, this rapid review addresses the following research questions:

1. What kind of digital methods and approaches have been used for the participatory development of health interventions?
2. What are the reported benefits and barriers of these digital participatory approaches?

## 2 Method

We conducted a rapid review following recommendations from the Cochrane Rapid Reviews Methods Group (14). Rapid reviews synthesize evidence using systematic methods, however, certain parts of a systematic review are either streamlined or omitted entirely, and they can be narrower in scope (15). As a result, rapid reviews are usually more resource-efficient and allow for a quicker synthesis of research compared to systematic reviews (16). The reporting of our methods and results was guided by the Preferred Reporting Items for Systematic Reviews and Meta-Analyses (PRISMA) guidelines (17).

### 2.1 Protocol development

The study protocol was drafted and presented to five community stakeholders to ensure that the planned review corresponds to stakeholders’ interests and obtain their input. The stakeholders (two working in healthcare, one in medical informatics, and two in community administration) were non-randomly selected from our professional network. Each received a digital copy of the review proposal and was invited to comment. After approximately one week, feedback was gathered in one-to-one meetings (three face-to-face, two per telephone). The stakeholders responded positively to the research questions. They were interested in the results, as they urgently sought digital solutions to engage with community members and address public (health) issues, particularly during the pandemic. No further changes to the research proposal were suggested. We next registered the review at the international prospective register of systematic reviews (PROSPERO, CRD42023387296).

### 2.2 Inclusion and exclusion criteria

We set the following eligibility criteria. First, studies had to report the development of a health-related intervention utilizing digital formats in the participatory process. Arnstein’s ladder of citizen participation (18) was used to estimate the level of participation. More specifically, studies were only included if their study process matched rung six (partnership), seven (delegation), or eight (citizen control) of the ladder. Research reporting lower levels of participation, for instance, consulting with community members through surveys (rung four on Arnstein’s ladder of participation), were excluded as they do not reflect thorough participation in which participants have a degree of decision-making power. Following the review by Schroeer et al. (6), studies were only included if digital formats were used in all or the main parts of the participatory intervention development process. We excluded studies in which digital tools, such as digital cameras, were used as a pure replacement for their analog counterparts. Accordingly, we excluded several photovoice studies, in which photos were taken with digital cameras but afterwards printed out or screened with a projector and discussed during in-person meetings (e.g. 19–21). However, we included studies where photos were taken with digital cameras or cellphones, which were then further used in digital ways, such that they were, for example, presented or discussed online (22). We also excluded studies that replaced initially planned in-person meetings with video conferencing during the COVID-19 pandemic without using any additional digital elements (e.g. 23). Thirdly, we only included original research and excluded theory papers, editorials, commentaries, review papers, conference abstracts and other reports. Fourthly, we only included studies published in English due to time constraints and consistent with guidelines for Cochrane rapid reviews (14). Finally, studies had to be published from 2010 onwards. This is a typical cut-off used in reviews examining digital (participatory) methods, as digital formats from earlier years are likely to be outdated (6, 24).

### 2.3 Search strategy

We consulted with an information specialist from the State and University Library Bremen (SuUB), Germany, to develop our search strategy. In October 2022, one author (FD) systematically searched the databases PubMed, Embase, PsycINFO, and Cochrane CENTRAL. We used combinations of keywords that pertain to digital participatory interventions in the health domain. Our search strategy is detailed in the Supplementary material. We additionally screened reference lists of available reviews on digital participatory research (6, 12), however, no further studies meeting the inclusion criteria were identified.

### 2.4 Study selection

Studies identified during the literature search were downloaded and imported into the web application Rayyan (25) to remove duplicates and to conduct the screening process. The majority of the screening team (FD, IS, CR, RS, SR, KB) pilot-tested the screening criteria, with some criteria (e.g., digital formats employed) adjusted for clarity. Next, abstracts from 25% of the identified records were randomly selected and subsequently dual-screened by two authors (FD, IS). Any discrepancies in judgment were resolved through discussion between the two reviewers, with the option to consult with a third reviewer (KB) to reach consensus. In line with Cochrane guidelines for rapid reviews (14), all of the remaining titles and abstracts were screened by one author (FD). A second review author (RS) screened all excluded abstracts, again with the option to involve a third reviewer (KB) to resolve discrepancies in judgment. In the next step, the entire screening team (FD, IS, CR, RS, HB, KB) reviewed the same five articles (26–30) in full-text to test the full-text review form. Once the piloting was completed, one reviewer screened all full-text articles (FD). Following this, a second reviewer (KB) screened all included articles, while a third reviewer (SR) screened all excluded articles.

### 2.5 Data extraction and quality assessment

Data was extracted from the included full-texts into a piloted Excel form, which assessed author, year of publication, study year, country of study, health domain, goal of the study, a sample description (e.g., sample size, age, and gender distribution), description of the participatory process, the digital format and tools used, and reported benefits as well as barriers of the utilized formats. Following Cochrane recommendations for rapid reviews (14), one reviewer (FD) extracted all data from the included literature, and a second reviewer (KB) double-checked for completeness and accuracy.

Given that the primary focus of this review was to identify digital formats used in participatory intervention development rather than assessing the quality of, for example, the analyses, and considering the high heterogeneity of the studies and the reporting formats, we refrained from formally evaluating the quality of the included studies.

## 3 Results

**Figure 1:**
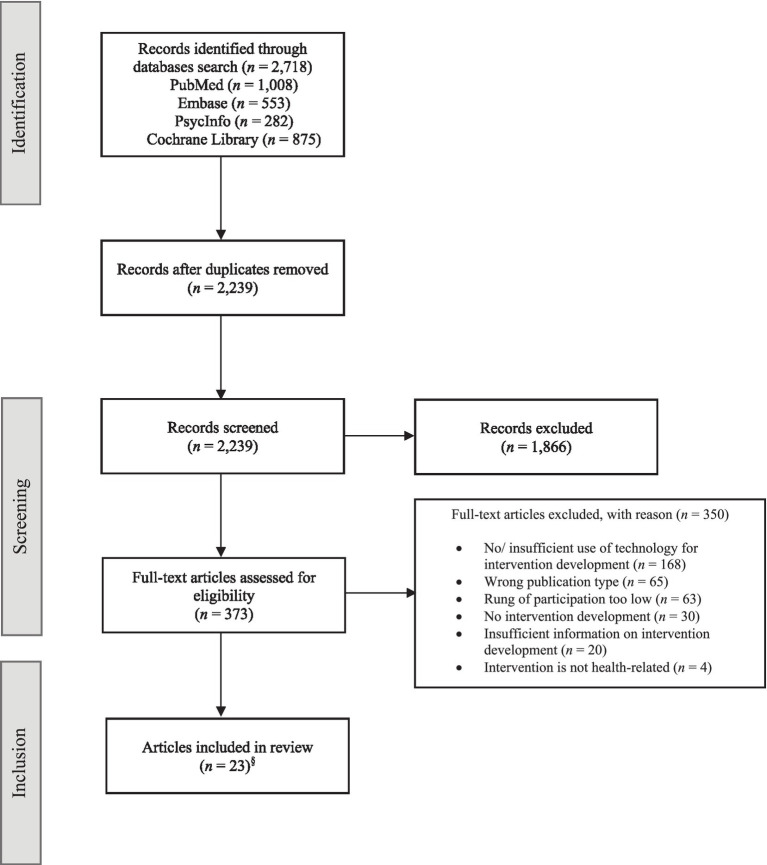
Flowchart. ^§^Two of the included articles report on the same study and were therefore combined for the analyses, resulting in a total of 22 studies in the review.

The database searches yielded 2,718 initial records. After removing duplicates, 2,239 titles and abstracts were screened. Of these, 373 records were retained for the full-text screening, during which 350 articles were excluded with reason (Figure 1). In total, 23 records met the inclusion criteria in this rapid review. Please note that the articles by Kennedy et al. (9) and Binder et al. (31) report on the same study and were therefore combined in the analyses. Hence, our review includes a total of 22 participatory health intervention studies.

### 3.1 Study characteristics

Table 1 provides an overview of the key characteristics of all included studies. The studies were published over the period spanning from 2010 to the beginning of 2023. The intervention development process of ten studies (45.5%) took place after the onset of the COVID-19 pandemic (9, 22, 32–39). Geographically, the research was distributed as follows: six studies were conducted in Canada (36, 37, 40–43), five in the US (32, 35, 44–46), three in Australia (9, 31, 38, 39), three in the UK (33, 47, 48), two in South Africa (34, 49), one in China (50), one worldwide (51), and one was conducted partially in Mexico and partially in the US (22).

**Table 1:** Characteristics of all included studies.

Citation	Study year	Study region	Health domain	Study aim	Target population	Sample (*n*, gender, age)
Arevian et al. (44)	2015–2016	USA	Mental health	Co-creation of a mobile texting app	Patients and providers in a clinical setting	Patients with obsessive compulsive disorder in an intensive outpatient program [*n* = 28; 39% female, 18–69 years, (*M* = 33, SD = 14.2)] Providers (*n* = 4)
Birkenstock et al. (32)	n/a	USA	Mental health	Needs assessment	Immigrant adolescents	Immigrant adolescents (*n* = 5)
Blake et al. (33)	2020	UK	Health education	Co-creation of a COVID-19 vaccine multimedia e-learning package	Healthcare workers and trainees	Discussion groups: Nurses (*n* = 28; 86% female); Healthcare students (*n* = 20; 55% female, 18–42 years) Storyboarding: Nurses (*n* = 5), Other healthcare professionals (*n* = 16), Members of the general public (*n* = 3) Project team: A health psychologist, an occupational health nurse, and a technologist (*n* = 3) Peer review of content and technical development: Experts (*n* = 23)
Chavez-Baray et al. (22)	2021	Mexico/USA	Health care	Needs assessment	Migrant transgender women of color	Migrant transgender women of color [*n* = 16; 18–45 years, (*M* = 27)]
Dada et al. (34)	2020	South Africa	Health education	Co-creation of accessible health education materials	Youth with severe communication disabilities	Young adults with severe communication abilities using augmentative and alternative communication (*n* = 6; 50% female; 24–34 years)
Fletcher et al. (40)	2012–2014	Canada	General health	Co-research on health promotion	Aboriginal youth	Youth (co-)research team: Adolescents and young adults (*n* = 8; 13–25 years) Workshop participants: Youth (approx. *n* = 230); Elders from First Nations communities (*n* = 14)
Hildebrand et al. (51)	2011	Worldwide	HIV/AIDS	Co-creation of a youth-led policy strategy	Youth	Adolescents and young adults from 79 countries (*n* = 3,479; 15–29 years)
John et al. (45)	n/a	USA	Health behavior	Co-creation of community-based health promotion programs	Rural communities	Rural communities in six Western US states [*n* = 21; populations 415–9,055 (median = 1,905) involving around 600 residents]
Kennedy et al. (9)/Binder et al. (31)	2020	Australia	Mental health	Co-creation of a mental health program	Primary producers	Primary producers (*n* = 12) Other stakeholders (*n* = 11)
Kornfield et al. (35)	n/a	USA	Mental health	Co-creation of an automated text messaging tool	Young adults with mental health concerns who are not in formal treatment	Young adults with at least moderate levels of depression or anxiety symptoms: Discussion group [*n* = 22; 81% female; 18–25 years (*M* = 21.5)]; co-design group (*n* = 9; 78% female)
Libon et al. (36)	2021	Canada	Mental health	Co-design of a mental health promotion program (prototype level)	Youth in a rural setting	Adolescents and young adults from small rural communities [*n* = 11; 15–24 years (Median = 20)]
MacEntee et al. (37)	2017–2019	Canada	General health	Case study on new method (quilted cellphilm method) for needs assessment in marginalized communities	Female sex workers	Sex workers (*n* = 15; 100% female; 19–25 years)
Odunitan-Wayas et al. (49)	2018	South Africa	Health behavior	Needs assessment	Residents of the Khayelitsha township	Participants of an ongoing study in the township who were either physically active or interested in increasing their physical activity [*n* = 11; 73% female; 21–25 years (*M* = 34.5, *SD* = 7.6)]
Ravalier et al. (47)	2019–2020	UK	Mental health	Co-creation of well-being interventions	Social workers	Needs assessment and intervention development with social workers [semi-structured interviews (*n* = 19); four focus groups (each *n* = 8)] Intervention refinement with senior organizational representatives (*n* not stated)
Ravalier et al. (48)	2018–2020	UK	Mental health	Co-creation of mental health and well-being interventions and improvements of working conditions	Health care workers	Needs assessment and intervention development with social workers [semi-structured interviews (*n* = 20); four focus groups] Action learning group consisting of key stakeholders, including senior management, staff, and union representatives (*n* = 13)
Sanchez-Pimienta et al. (41)	2020	Canada	General health	Co-research on health perception using digital storytelling	Indigenous youth	Indigenous adolescents and young adults (*n* = 5; 17–22 years)
Sheats et al. (46)	2013–2014	USA	Health behavior	Needs assessment	Older adults	Older adults aged 60 years or more and were able to move unaided [*n* = 23; 70% female; 61–92 years (*M* = 70.8, SD = 7.7); 39% Hispanic/Latino, 26% Asian, 13% White, 9% Black, 9% Hawaiian/Pacific Islander, 4% Native American/ Alaskan Native]
Shemesh et al. (38)	n/a	Australia	Cancer	Co-creation of a web-based portal for self-management of patient-related outcomes of prostate cancer	Men with prostate cancer	Men with prostate cancer from a previous project on prostate cancer (*n* = 28) Health professionals treating, managing, or supporting men with prostate cancer (*n* = 39)
Snider et al. (42)	2009	Canada	Violence prevention	Co-design of a hospital-based intervention, including indicators for evaluation	Youth	Brainstorming session: *n* = 48 (40 adolescents, 1 parent, 5 community youth workers, 2 others / did not reveal) Sorting session: *n* = 103 (60 adolescents, 20 parents, 17 community youth workers, 6 others / did not reveal) Rating session: *n* = 102 (60 adolescents, 20 parents, 16 community youth workers, 6 others / did not reveal) Interpretation session: *n* = 25
Tang et al. (50)	2016–2017	China	HIV/AIDS	Co-creation of an HIV testing intervention	Men who have sex with men (MSM)	Nationwide contest: Entries by general Chinese public (*n* = 431); Judges: local MSM and experts Regional strategy contests (analog)
Tay et al. (39)	2020	Australia	Health behavior	Co-creation of an app-based dietary intervention	Adults at risk of type 2 diabetes	Adults (*n* = 20; 5 pre-diabetes, 9 diabetes, 6 not specified); clinical experts (*n* = 4)
Whitley et al. (43)	2015–2019	Canada	Mental health	Co-creation of documentary-style videos to educate and reduce stigma	General population	People in recovery from severe mental illness (schizophrenia, bipolar disorder, major depression or schizoaffective disorder) The Montreal site: *n* = 10 (6 men, 4 women); Halifax: *n* = 9 (4 men, 5 women). Aged between 20–64 years The Toronto site: *n* = 4 (3 men, 1 women), all under age 30

The spectrum of health domains covered by these studies was notably diverse. Specifically, eight studies (36.4%) centered on mental health and well-being (9, 32, 35, 36, 43, 44, 47, 48), examining aspects such as stress management (48), suicide prevention (36), and mental health stigma (43). Four studies (18.2%) addressed health behaviors (39, 45, 46, 49), such as obesity prevention (45) or physical activity promotion (49). Another four studies (18.2%) delved into the broader domain of general health (37, 40, 41) and healthcare (22). Health education, including themes concerning COVID-19 prevention and vaccination, was the subject of two studies (33, 34). Another three (13.6%) targeted specific diseases, namely HIV/AIDS (50, 51) and cancer (38). One (4.5%) fell into the domain of youth violence prevention (42).

The majority of the included studies (*n* = 14, 63.6%) targeted adults (9, 22, 33, 37–39, 43–50). Eight studies (36.4%) targeted youth or young adults (32, 34–36, 40–42, 51). Only one study targeted older adults (46). The number of participants involved in the intervention development process ranged between *n* = 5 and *n* = 3,479.

### 3.2 Digital participatory formats

An overview of the digital participatory approaches employed by the included studies, along with information on their participatory elements, technical facilitators, and reported benefits and barriers, is presented in Table 2. Overall, we identified three groups of digital participatory formats employed, which will be detailed in the following.

**Table 2:** Digital participatory approaches.

Citation	Digital format used for participatory elements	Digital tools employed	Technical facilitators	Reported benefits format	Reported barriers format
Web-based participatory approaches					
Arevian et al. (44)	App co-creation (content and functionality) via a web-based application development platform (Chorus)	Chorus platform	1-h training for the psychotherapists on use of the platform	Platform allows to create apps without programming skills	n/a
Birkenstock et al. (32)	3 online art workshops with professional artists and designers using various forms of digital artwork (e.g., photos, videos, memes, music); Co-creation of a digital zine	Unspecified videoconference software	Instagram direct messages for communicating programmatic changes and keeping connected	n/a	Internet access required; Fatigue from being online additionally to online school; Switched-off cameras reduced sense of connectedness; Simultaneous family obligations (e.g., care work) reduced involvement in discussions
Blake et al. (33)	2 online group discussions to define topic, learning outcomes, target group; Asynchronous virtual storyboarding for content, design, and key messages	Microsoft Teams	Storyboarding with prepared resources but without real-time facilitators	n/a	n/a
Dada et al. (34)	Asynchronous online group discussions; Synchronous webinar for result presentation	WhatsApp group; Unspecified webinar software with audio-reading and chat function	n/a	Communication technology facilitated participation of persons with severe disabilities when in-person contact was not possible	Challenges with internet connectivity
Hildebrand et al. (51)	9 open online forums to generate and vote on solutions; Online sessions to draft final strategy document; Online dialog to co-author final strategy document	Facebook, RenRen, and Vkontakte with voting functionality; GoogleDocs; Unspecified software for online dialog	Community mobilizer facilitated online forum discussions	Reaching a large number and wide range of young people worldwide; Overcoming digital divide through a mix of online and offline fora	n/a
Kennedy et al. (9)/Binder et al. (31)	2 online co-design workshops (Password-protected)	Zoom platform with breakout rooms, live polls; live on-screen notes; click and drag to rank activity cards; chat function	One facilitator per breakout room; One person for documentation and support	Opportunity to access hard-to-reach groups and geographically scattered populations; Resource efficient, saving costs for, e.g., travel; Online co-design reduces power imbalances Smaller breakout rooms: More comfortable environment, establish rapport, help people with difficulties with social interactions to participate	Requires facilitators for participants with insufficient online expertise; Internet connection might be poor in rural areas; Online co-design can produce more data when multiple breakout rooms are active at the same time; Zoom fatigue can be an issue
Kornfield et al. (35)	Online asynchronous discussion group (pseudonymized) with prompts; 5 online co-design workshops (anonymous, optional video-function)	Study platform; Zoom workshops with screen-sharing	Two persons for the workshops	Asynchronous discussion group is flexible and convenient; Remote and anonymous participation	People who participated might have been more motivated
Libon et al. (36)	3 online co-design workshops	Zoom platform with breakout rooms	n/a	n/a	Requires access to private and stable internet connections
Ravalier et al. (47)	4 online group discussions	GoToMeeting platform with virtual whiteboard	n/a	n/a	n/a
Ravalier et al. (48)	4 online group discussions	GoToMeeting platform with virtual whiteboard	n/a	n/a	n/a
Shemesh et al. (38)	4 online group discussions; 7 online co-design workshops	Zoom platform with polling; MURAL’s platform with several unspecified functions	n/a	Online nature may have benefitted attendance rate and participants’ diversity (reduced geographical barriers, adherence to social distancing measures) Online environment increased anonymity may have made it easier to share experiences and issues	n/a
Snider et al. (42)	Asynchronous online brainstorming (8 weeks accessible); Asynchronous online sorting, rating, and labeling (6 weeks accessible)	The CS Global software (Concept Systems, Ithaca, NY)	n/a	Anonymous participation was especially valuable, given the sensitive subject; Participants could engage in any or all study parts	n/a
Tang et al. (50)	Crowdsourcing through a nationwide open contest for images and concepts	n/a	n/a	Inclusive and efficient approach to gathering community input	n/a
Tay et al. (39)	3 online co-design workshops (anonymous contribution options)	Zoom platform with break-out rooms, Miro app (virtual sticky notes), Mentimeter app (polling)	Before workshops: Platform practice activities and online drop-in sessions; During workshops: Trained facilitators	Efficient and less time-consuming method; Overcomes geographical and mobility barriers	Still requires considerable preparatory work and operational work of facilitators; Online format might have prevented participation in some cases among others due to ease of not attending the online format.
Digital participatory visual approaches					
Chavez-Baray et al. (22)	Photovoice with phone cameras (for 4 weeks); 4 online synchronous group meetings for development of recommendations and strategies	Unspecified phones for photo taking; Zoom platform	n/a	n/a	n/a
Fletcher et al. (40)	Digital storytelling; Training and weekly meetings through webinars and conference calls; Communication through messenger	iMovie to create and edit digital stories; Unspecified webinar, conference call, and messenger software	Youth and media students aided participants in creating their digital stories in iMovie Mobile media van to reach rural communities for workshops	Digital storytelling method is highly accessible and engaging; Creating digital stories provided youth with opportunities to reflect own experiences	Technical quality of digital stories was dependent on whether participants were able to complete the story during university sessions or on technical skills of the participant
MacEntee et al. (37)	Quilted cellphilm method: Short individual videos were made with (cell-)phones and cut into a composite video	Researchers’ or private phones for creating the short videos;iMovie for movie-editing	Facilitators for creating and editing the individual videos	Method can be used without formal media training; Revisions and edits are easily done	n/a
Sanchez-Pimienta et al. (41)	Digital storytelling	n/a	Participants were taught about video scripting, filming, and editing	n/a	n/a
Whitley et al. (43)	Participatory video method: Sessions over 18-month period, during which 26 documentary-style videos were created; Dissemination via project website and YouTube channel	n/a	3 professional videographers (one per site) who organized training sessions	Videos enhance credibility (“seeing is believing”); Cost-effective esp. for remote areas and wider reach through digital method; Anonymity is beneficial for sensitive topics	n/a
Digital participatory mapping					
John et al. (45)	Digital participatory photomapping to document environmental attributes that affect dietary behavior and physical activity	Camera-enabled GPS units (provided to the participants) ArcGis 10.1 for mapping the photos	3-day on-site workshops for facilitators; Participants received training on how to use the GPS units	Method enhances understanding, encourages community engagement, and prompts evidence-based actions	Sometimes, community characteristics well-known to locals were not photographed Barriers were minimized by providing transportation, gasoline, and / or bilingual support as needed
Odunitan-Wayas et al. (49)	Digital participatory mapping to document usual routes for physical activities as well as enablers and barriers	The Stanford Healthy Neighborhood Discovery Tool (an app for smartphones or tablets collecting various data, including GPS-tracked walking routes, geo-tagged audio stories, and images)	Participants received training on Discovery Tool; Facilitators joined participants during Discovery walks	Can empower low SES community members; Offers opportunities for citizens to collaboratively analyze and prioritize information and to suggest potential solutions	Missing data caused by poor internet connection when uploading the collected data to the protected server
Sheats et al. (46)	Digital participatory mapping to document a shopping trip to a typical food store	The Stanford Healthy Neighborhood Discovery Tool (see above)	Participants received training on the Discovery Tool; Facilitators joined participants during Discovery walks; In-app reminders to take photos and record audio narrative	n/a	n/a

#### 3.2.1 Web-based digital participatory formats

Most of the included studies (*n* = 14, 63.6%) used web-based digital participatory formats, encompassing participatory approaches that utilize online communication platforms, such as Zoom or WhatsApp, or various online applications and their features in the intervention development process. These web-based digital participatory formats have *general* benefits and barriers. One highlighted advantage is that they provide a high level of anonymity, which is particularly valuable in the case of sensitive topics (35, 38, 42). Shemesh et al. (38), for example, note that the increased anonymity of an online environment might have made it easier for their participants, who were men with prostate cancer, to open up. Similarly, Snider et al. (42) acknowledge that the formats’ possibilities for anonymous participation were particularly valuable for the sensitive topic of their study on youth violence. Another general benefit of web-based participatory formats reported in several papers is the opportunity to access hard-to-reach groups and overcome geographical barriers (9, 39), thereby having more participant diversity (38). Moreover, the formats’ resource efficiency, saving costs and time for travel to meeting places, is mentioned as an additional advantage (9, 39). Notably, the formats are not without limitations. One general issue raised by several of the included papers is the formats’ dependency on a stable internet connection, excluding persons who do not meet this prerequisite and making it potentially more difficult to fully participate for those living in more rural areas (9, 32, 34, 36).

One important distinction among web-based digital participatory formats is their synchronous or asynchronous nature (6, 12). In synchronous web-based participatory formats, participatory intervention development activities occur in real time, usually through online platforms or video conferencing software. In contrast, asynchronous formats, such as online forums, do not require real-time interaction or immediate responses. In the present review, eight studies used synchronous web-based participatory formats (9, 32, 36, 38, 39, 44, 47, 48). In one study, psychotherapists and their patients collaboratively developed a mobile texting application to support therapy using an online application development platform (44). Following a brief training session for the therapists, participants were able to co-create the applications due to the platform’s user-friendly interface. However, most web-based participatory synchronous studies conducted online discussions or online co-design workshops utilizing online video conferencing software. For instance, Kennedy et al. (9) conducted co-design workshops to develop mental health promotion strategies among producers from the primary sector in Australia. Similar to other projects (39), they used various digital features of their video conferencing tools during their workshops. These include breakout rooms to split up the group for smaller discussions and activities, live polls to vote on ideas and solutions, on-screen notes, and the chat function for questions and feedback. Another study conducted participatory online art workshops with youths, allowing them to express their mental health-related experiences and needs through diverse forms of digital artwork, such as memes or music (32).

Benefits reported from conducting synchronous co-design workshops and group discussions on online platforms like Zoom include the option to incorporate features like small breakout rooms, which help foster rapport with participants and encourage active engagement, particularly for individuals who may face challenges with social interactions (9). Apart from that, it is argued that the format can mitigate some power imbalance in research, as researchers and participants are in the same online space and might be challenged by the same issues associated with the format (9). The synchronous format was also a viable option for continuing co-design sessions when in-person meetings were not possible (34).

Despite the aforementioned benefits, the synchronous formats also come with several barriers. Two studies stated that online fatigue may have been an issue (9, 32). For instance, some youths participating in the study by Birkenstock et al. (32) reported that they were tired from being online at school due to the pandemic and additionally attending online workshops as part of the study. Also, active participation in discussions was hampered for some participants who simultaneously had to fulfill family obligations, such as caring for younger siblings (32). Another issue raised is a reduced sense of connectedness while meeting online with switched-off cameras (32). One study pointed out that the participation rate was probably reduced as it is relatively easy to cancel online meetings (39). For researchers, having multiple break-out rooms implies having a lot of data, which was mentioned as a disadvantage (9). Finally, even though some organizational tasks associated with in-person meetings become redundant, this format still requires considerable preparatory work and technical facilitation to assist less computer-literate participants (9, 39).

Asynchronous formats, which do not require real-time interaction or immediate responses, were employed in three studies. One of these studies conducted online concept mapping, which entailed asynchronous online tasks for generating and rating ideas regarding youth violence prevention (42). More specifically, participants were asked to engage in an online brainstorming task, which remained accessible for eight weeks. They later had the option to sort and label the collected ideas (available for six weeks). The outcomes were discussed during an in-person community meeting. Snider et al. (42) underline the freedom to participate in either activity or both as a benefit of this format. Another asynchronous crowdsourcing approach was taken by Tang et al. (50), who organized a nationwide contest to collect images and concepts promoting HIV testing. The authors note that community input can be gathered inclusively and efficiently through this approach (50). In another study, asynchronous online group discussions were conducted using a WhatsApp group for the collaborative development of COVID-19-related health materials involving youth with severe communication disabilities (34).

Three studies employed a combination of synchronous and asynchronous formats (33, 35, 51). The study by Blake et al. (33) first held synchronous discussion groups on Microsoft Teams to establish the aims of a learning package on the COVID-19 vaccine but subsequently conducted asynchronous online storyboarding to draft the package’s content. The other two studies that used this hybrid approach started with an asynchronous format and later followed with a synchronous format (35, 51). Hildebrand et al. (51) involved over 3,000 young adults worldwide in an asynchronous crowdsourcing exercise on various social media platforms, asking them to generate and vote on solutions regarding the integration of youths’ perspectives into AIDS policies. Discussions were facilitated by community mobilizers. Later, synchronous public online sessions took place to draft the final strategy document. Noteworthy, this study also held 39 offline forums worldwide to mitigate the digital divide. Kornfield et al. (35) first conducted asynchronous online discussions with pseudonymous accounts on the study’s platform. Prompts with several questions related to mental health and how a messaging application could promote mental health were regularly released. The authors highlight the flexibility and convenience of asynchronous discussion groups (35). In addition to the discussion group, five synchronous online co-design workshops on Zoom were hosted, where participants could further refine the mental health application. Importantly, participation was anonymous (first names only), with the option to turn off the video function. None of the studies directly addressed the benefits or drawbacks of their combined approach.

#### 3.2.2 Digital participatory visual formats

Five studies (22.7%) employed digital participatory visual methods, including photo-and videovoice, digital storytelling, and participatory video. These formats allow participants to document their experiences and reflect on their perspectives through means of photos or videos. One study, for example, conducted digital photovoice with migrant transgender women of color (22). Over a four-week period, participants used cellphones to capture their daily experiences and challenges. In addition, online group and in-person meetings were held to share and discuss the photos, and ultimately, a call for action was developed (22).

Participatory video, which typically enables workshop participants to actively collaborate in planning, filming, and sometimes implementing the public screening of the resulting videos (52), was utilized by two studies (37, 43). In a study by Whitley et al. (43), participants in recovery from a severe mental illness engaged in participatory video sessions and created 26 documentary-style videos with the aim to reduce mental health stigma. These were presented on a YouTube channel and during public screenings. Another study by MacEntee et al. (37) utilized a unique form of participatory video referred to as ‘quilited cellphilm method’. This format involves participants planning and creating short (< 5 min) individual films with their cellphones, addressing a prompt or question. The individual films are later combined into a composite video (41). In the study by MacEntee et al. (37), video-making and editing were assisted by facilitators. Both articles mention some benefits of their respective participatory video format. Whitley et al. (43) emphasize the method’s ability to ensure participant anonymity, making it particularly well-suited for addressing sensitive topics. They further note the cost-effectiveness of the approach for remote areas and its capability for a wide reach due to its digital format. Another reported benefit the authors mention is that the resulting videos can enhance the credibility of the issue they address (43). Reported benefits of the cellphilm method taken by MacEntee et al. (37) included that the method can be used by participants without formal media training and that revisions and edits are relatively easily done. Barriers were not reported by either of the included studies.

Another digital participatory visual format included in the review is digital storytelling, which involves ‘3-to 5-min visual narratives that synthesize images, video, audio recordings of voice and music, and text to create compelling accounts of experience’ (53), p.186. One included study adopted a participatory video approach using both analog and digital methods. Participants were taught about video-making and editing, along with training on ethics and leadership skills so that they could eventually take over the project (40). Another co-research study with indigenous youth was included in this review, where participants chose participatory video as a method and received elaborate one-on-one assistance in scriptwriting, storyboard design, media gathering, and video editing (41). Concerning the benefits of the format, Fletcher and Mullet (40) emphasize that the method is highly accessible and engaging. Creating digital stories also provided youths with opportunities to reflect on their own experiences. However, one drawback of the format reported by these authors is that the technical quality of the stories depended on whether participants completed editing their stories during a university session and on the technical skills of the participants (40).

#### 3.2.3 Digital participatory mapping

Three of the included studies (13.6%) employed digital participatory mapping formats (45, 46, 49), which encompassed the use of Geographic Information Systems (GIS) or Global Positioning Systems (GPS) techniques to collect, visualize, and analyze spatial data that affect community health. The study by John et al. (45) used digital participatory photomapping to document food environments. More specifically, the study used participatory photomapping with GPS technology to assess communities’ readiness as well as barriers to healthy eating and physical activity, followed by focus groups and community discussions, among other things. To ensure the successful run of the study, local facilitators were trained for three days, which in turn delivered training to participants on how to use the GPS units. The authors state that the format can enhance understanding and motivate community engagement and evidence-based changes within communities (45). However, they also note that some community characteristics were not photographed although regularly encountered. The authors also report that they mitigated potential barriers to participation by providing transportation, gasoline, and, if required, bilingual support. In two other studies (46, 49) digital participatory mapping was conducted using the Stanford Healthy Neighborhood Discovery Tool (54), which is available as a mobile app for Android and Apple iOS smartphones and tablets. The tool not only tracks walking routes but also geo-tagged audio narratives and photos. Odunitan-Wayas et al. (49) used the Discovery Tool in a South African community and asked participants to take their daily walking route and document barriers as well as facilitators for physical activity. Prior to the walks, participants received training on the tool. During their walks, they were accompanied by a facilitator to ensure assistance and safety. Sheats et al. (46) also employed the Discovery Tool and invited low-income and food-insecure older adults to document their food environment and to identify their needs concerning food selection and purchase. Similar to the study by Odunitan-Wayas et al. (49), participants were trained by facilitators and accompanied during the walk for assistance. With regard to the reported benefits of the Discovery Tool, Odunitan-Wayas et al. (49) stress its potential to empower low socioeconomic status community members as well as provide opportunities for community members to collectively analyze data and develop solutions. However, they also noted that missing data attributed to poor internet connections during data uploads to a protected server can be an issue.

A summary of the reported input, reported benefits and barriers of the three types of formats is presented in Table 3.

**Table 3:** Summary of digital participatory formats and their reported benefits and barriers.

Digital participatory format	Reported inputs	Reported benefits	Reported barriers
Web-based digital formats	Technical facilitators to train and assist with the use of online platforms and online activities	Facilitates participation of persons with severe disabilities Wide reach, including reach to geographically dispersed persons Resource-efficient, saving time and costs, e.g., for travel, catering, room bookings Can encourage the participation of persons with social interaction difficulties Anonymous participation is possible, which is especially beneficial for sensitive topics Asynchronous formats allow participants to align participation with their schedule	Internet connectivity might be esp. problematic in socially disadvantaged groups and rural areas Online fatigue Risk of reduced sense of connectedness Potential distractions from household members when participating from home. May require technical facilitators Multiple simultaneous online breakout rooms generate more data People who participate might have been particularly motivated Requires substantial preparatory and operational work from facilitators Barrier to canceling participation in online meetings may be lower than compared to in-person formats
Visual formats	Provision of phones (if not relying on participants’ private phones) Provision of film editing software Technical facilitators to train and assist with creating and editing visual content	Wide reach also of geographically dispersed persons Resource-efficient, saving time and costs, e.g., for travel, catering, rooms Allows for anonymous participation Engaging and creative Provides participants with an opportunity to share and reflect on personal experiences Enhanced credibility (“seeing is believing”) Some formats may be done without formal media training with simple editing options	Requires a certain level of technical skills, which may be challenging for some groups (e.g., older adults)
Mapping formats	GPS units for the participants Technical facilitators to train and assist in using GPS units	Engages communities May empower low socio-economic status community members Provides opportunities for community members to collaboratively analyze, prioritize, and suggest potential solutions to issues	Participants may not document well-known characteristics of their community May require provision of additional resources, such as transportation, gasoline, or bilingual support Missing data due to poor internet connectivity during data upload

## 4 Discussion

The present review included 22 studies that employed digital formats for the participatory development of health interventions which could be categorized into three types of formats: web-based participatory formats, digital participatory visual formats, and digital participatory mapping.

Across formats, various benefits and barriers were reported. Several web-based studies (35, 38) and digital participatory visual formats (43) highlighted the possibilities for heightened participant anonymity of digital participatory approaches. These formats were, therefore, considered particularly well-suited for sensitive topics (42). Apart from that, several authors underscored the wide reach of digital formats, enabling the crowdsourcing of ideas from diverse populations across countries (51) and facilitating access to hard-to-reach groups that are geographically dispersed (9). Digital participatory visual approaches and digital participatory mapping, in particular, were described as highly engaging for community members (45), especially for youth (40). An additional reported advantage for both researchers and participants was resource efficiency, saving time and expenses, for example, related to travel to research destinations (9).

It was also highlighted that although digital participatory intervention development can save time and costs, it does require extensive preparatory work and the presence of facilitators (39). Another common barrier reported across formats was a required stable internet connection to participate in web-based studies (32, 36) and to reliably upload collected data to protected servers and thus avoid loss of data in digital participatory mapping (49). Notably, some barriers discussed in the literature were missing in the publications included in this review. For instance, discussions on how the digital setting affected feelings of connectedness among participants were largely absent. Only one study reported on issues regarding sense of connectedness, which, in that study, was attributed to switched-off cameras (32). Another study mentioned the relatively low psychological barrier to canceling online meetings (39). In line with findings from a review of digital participatory formats by Schroeer et al. (6), concerns regarding participants’ privacy and data protection were also not discussed in the literature included in this review. Synchronous web-based formats, such as online workshops or discussions, in particular, may raise ethical issues, such as disruptions during meetings with sensitive topics by other household members or participants secretly recording sessions (12). It has been recommended to disclose these issues in the consent and emphasize the importance of confidentiality (12).

Similar to findings from Schroeer et al. (6), the results of this review furthermore indicated that digital participatory intervention studies predominantly targeted (younger) adults, with only one study focusing on older adults (46). This discrepancy suggests that participatory intervention designs involving technology might be perceived as less suited for older adults, who, despite an increase in technology use in the last years, persist in having lower internet access and usage (55), and generally possess lower computer skills (56). This raises questions pertaining to how inclusive digital participatory intervention development can be. Previous research has explored how digital participatory research methods, such as digital photovoice or discussion groups, can be employed with people who are not technical savvy (57) or who live in rural communities (23). Moreover, in many of the included studies training was offered or facilitators were present who assisted participants with issues regarding technology during the conduct of the study (39), and one study even offered both on-and offline forums (51). Thus, in the included literature several efforts were made by researchers to overcome disparities in technological skills. However, although this is not the focus of the present review, it is important to consider what measures researchers can take at earlier stages, particularly during recruitment, to bridge the digital divide and foster inclusion in digital participatory studies (58).

While the included studies were highly heterogeneous with regard to their digital format, it is noteworthy that many of the included digital formats are based on analog approaches that have been long established in participatory research. For example, analog participatory mapping, also known as hands-on mapping, where participants create maps on, for instance, paper, has been frequently used in public health research (59). However, the digital formats might bring added value to their analog counterparts. Boerner et al. (60) argue that in participatory (action) research, limited attention has been paid to the question of how digital participatory approaches can be exploited to better include marginalized groups. The digital tools used in some of the included studies may well allow participants to express themselves more freely (38). Studies that, for instance, offer participants to switch off their cameras during discussion groups (35) can increase anonymity beyond what would be possible in most face-to-face formats. Notably, innovative digital concepts, such as virtual reality technology were only used as end products (61), but not for the participatory development of health-related interventions. Once technical innovations, such as virtual reality headsets, become more accessible and affordable, future studies might also test and use them as tools for participatory intervention development.

### 4.1 Strengths and limitations

This rapid review provides a comprehensive overview of existing digital participatory formats following guidelines by the Cochrane Rapid Reviews Method Group (14), yet, it is not without limitations. While we consulted with an information specialist to identify keywords that should ideally enable us to retrieve all relevant studies, we might have missed several studies. First of all, due to the time constraints of a rapid review, we only searched a limited number of databases, in our case four, restricted the literature’s language to English and omitted grey literature, as well as supplemental searches. Moreover, the heterogeneity in terminology regarding participatory approaches across countries (62) and disciplines could have resulted in missing some relevant studies. Although we used a diversity of common terms, we might have missed publications that used digital participatory approaches but deviated from that terminology. It is also important to acknowledge that the lack of a shared terminology and the lack of shared reporting standards made the literature screening process challenging. For this reason, a large number of articles had to be included in the full-text screening as it was often unclear from the abstract alone if the study took a participatory approach. While reporting standards have been developed previously (63), they have not been widely established among researchers yet; an issue that has also been acknowledged in other reviews of the participatory literature (64). A shared terminology and standardized reporting of participatory approaches would greatly facilitate the effective communication of research findings and assist in identifying relevant articles and synthesizing findings. This also applies to the terminology regarding the use of technology in participatory research. During the literature screening, it often remained unclear if the intervention development process incorporated technology or if analog techniques were used to develop a *digital* health tool, or both. Another limitation of this review concerns the quality assessment of the included studies. Despite our initial intention to additionally conduct a formal quality assessment, this was not feasible due to the high heterogeneity in the design and reporting of the included literature. Noteworthy, many of the included studies relied on small, non-random samples with a high risk for considerable selection bias. Moreover, while we only included studies where participants had decision-making power during the intervention development, we did not assess the extent to which participants could decide on the topic and method used to develop the intervention since this was not the focus of this review. Finally, it is important to acknowledge that the present review did also not assess the content and effectiveness of the developed interventions. While this was beyond the scope of the current review, an important task for future research is to assess in what aspects interventions developed through analog versus digital methods may differ.

### 4.2 Conclusion

This rapid review included 22 studies and provides an overview of digital participatory approaches to develop health interventions, which can be categorized into web-based, digital visual, and digital participatory mapping formats. These digital formats have benefits such as heightened anonymity, expanded reach, and enhanced cost-and time-efficiency compared to their analog counterparts. However, they also come along with challenges related to internet connectivity and technical proficiency, which may introduce selection bias. While researchers have addressed some of these challenges through measures such as technical facilitation, they persist as barriers to inclusive participation. The results of this review further revealed that many of these digital formats largely mirror their analog counterparts, underscoring the opportunity for more innovative approaches to participatory intervention development that exploit the participatory potential of technological developments. This review has also highlighted the need for shared terminology and standardized reporting standards within the field of digital participatory health intervention studies to enhance effective communication, synthesis, and generalizability.
