# Supplementary material for: A rapid review of digital approaches for the participatory development of health-related interventions
Source: Front Public Health. 2024 Nov 29;12:1461422. doi: 10.3389/fpubh.2024.1461422 (PMC11638186; doi:10.3389/fpubh.2024.1461422)
Supplement: Supplementary file 1 [file Table_1.docx]

Supplementary Material

A Rapid Review of Digital Approaches for the Participatory Development of Health-Related Interventions

**Friederike Doerwald, Imke Stalling, Carina Recke, Heide Busse, Rehana Shrestha, Stefan Rach, Karin Bammann***

*** Correspondence:** Karin Bammann: bammann@uni-bremen.de

**Pubmed (MEDLINE)** **Search Strategy**

| # | Searches |
| --- | --- |
| 1 | participatory research[tw] |
| 2 | community participation[tw] |
| 3 | participatory design[tw] |
| 4 | "action research"[tw] |
| 5 | co-design[tw] |
| 6 | codesign[tw] |
| 7 | co-creation[tw] |
| 8 | cocreation[tw] |
| 9 | co-research[tw] |
| 10 | OR (#1-9) |
| 11 | digital[tw] |
| 12 | online[tw] |
| 13 | Internet[tw] |
| 14 | virtual[tw] |
| 15 | social media[tw] |
| 16 | Smartphone*[tw] |
| 17 | mobile phone*[tw] |
| 18 | video*[tw] |
| 19 | photo*[tw] |
| 20 | OR (#11-19) |
| 21 | health research[tw] |
| 22 | health promotion[tw] |
| 23 | mental health[tw] |
| 24 | prevention[tw] |
| 25 | OR (#21-24) |
| 26 | (#10) AND (#20) AND (#25) |
| ALL | 2010:2022[pdat] |
| ALL | English[Filter] |

**EMBASE Search Strategy**

| # | Searches |
| --- | --- |
| 1 | participatory research/exp |
| 2 | community participation/exp |
| 3 | participatory design:ti,ab,kw |
| 4 | action research/exp |
| 5 | co-design:ti,ab,kw |
| 6 | codesign:ti,ab,kw |
| 7 | co-creation:ti,ab,kw |
| 8 | cocreation:ti,ab,kw |
| 9 | co-research:ti,ab,kw |
| 10 | OR (#1-9) |
| 11 | digital:ti,ab,kw |
| 12 | online:ti,ab,kw |
| 13 | Internet:ti,ab,kw |
| 14 | virtual:ti,ab,kw |
| 15 | social media[/exp |
| 16 | mobile phone/exp |
| 17 | video*:ti,ab,kw |
| 18 | photo*:ti,ab,kw |
| 19 | OR (#11-19) |
| 20 | health research:ti,ab,kw |
| 21 | health promotion/exp |
| 22 | mental health/exp |
| 23 | prevention/exp |
| 24 | OR (#21-24) |
| 25 | (#10) AND (#20) AND (#25) |
| ALL | 2010:2022[pdat] |
| ALL | English[Filter] |

**PSYCHINFO Search Strategy**

| # | Searches |
| --- | --- |
| 1 | participatory research (TI) OR participatory research(AB) OR participatory research (KW) |
| 2 | community participation(TI) OR community participation(AB) OR community participation(KW) |
| 3 | participatory design (TI) OR participatory design(AB) OR participatory design(KW) |
| 4 | action research |
| 5 | co-design(TI) OR co-design(AB) OR co-design(KW) |
| 6 | codesign(TI) OR codesign(AB) OR codesign(KW) |
| 7 | co-creation(TI) OR co-creation(AB) OR co-creation(KW) |
| 8 | cocreation(TI) OR cocreation(AB) OR cocreation(KW) |
| 9 | co-research(TI) OR co-research(AB) OR co-creation(KW) |
| 10 | OR (#1-9) |
| 11 | Digital(TI) OR digital(AB) OR digital(KW) |
| 12 | online(TI) OR online(AB) OR online(KW) |
| 13 | Internet(DE) |
| 14 | Virtual(TI) OR virtual(AB) OR virtual(KW) |
| 15 | social media[/exp |
| ~~16~~ | Smartphones(DE) |
| 17 | mobile phones(DE) |
| 18 | video*(TI) OR video*(AB) OR video*(KW) |
| 19 | photo*(TI) OR video*(AB) OR video*(KW) |
| 20 | OR (#11-19) |
| 21 | health research(TI) OR health research(AB) OR health research(KW) |
| 22 | health promotion/exp |
| 23 | mental health/exp |
| 24 | prevention/exp |
| 25 | OR (#21-24) |
| 26 | (#10) AND (#20) AND (#25) |
| ALL except MESH searches | With publication year from 2010 to 2022 |
| ALL except MESH searches | With Cochrane library publication date from Jan 2010 to present, in trials |

**Cochrane Library Search Strategy**

| # | Searches |
| --- | --- |
| 1 | participatory research:ti,ab,kw |
| 2 | Participatory research (MESH) |
| 3 | community participation:ti,ab,kw |
| 4 | Community participation (MESH |
| 5 | participatory design:ti,ab,kw |
| 6 | "action research":ti,ab,kw |
| 7 | co-design:ti,ab,kw |
| 8 | codesign[:ti,ab,kw |
| 9 | co-creation:ti,ab,kw |
| 10 | cocreation:ti,ab,kw |
| 11 | co-research:ti,ab,kw |
| 12 | OR (#1-11) |
| 13 | digital:ti,ab,kw |
| 14 | online:ti,ab,kw |
| 15 | Internet:ti,ab,kw |
| 16 | Interenet(MESH) |
| 17 | virtual[:ti,ab,kw |
| 18 | social media:ti,ab,kw |
| 19 | Social media(MESH) |
| 20 | Smartphone*:ti,ab,kw |
| 21 | Smartphone (MESH) |
| 22 | mobile phone*:ti,ab,kw |
| 23 | Cell phone (MESH) |
| 24 | video*:ti,ab,kw |
| 25 | photo*:ti,ab,kw |
| 26 | OR (#13-25) |
| 27 | health research:ti,ab,kw |
| 28 | health promotion:ti,ab,kw |
| 29 | Health promotion (MESH) |
| 30 | mental health:ti,ab,kw |
| 31 | Mental health(MESH) |
| 32 | prevention[:ti,ab,kw |
| 33 | OR (#26-32) |
| 34 | (#12) AND (#26) AND (#33) |
| ALL | 2010:2022[pdat] |
| ALL | English[Filter] |
